# Association of arterial blood pressure and vasopressor load with septic shock mortality: a post hoc analysis of a multicenter trial

**DOI:** 10.1186/cc8167

**Published:** 2009-11-16

**Authors:** Martin W Dünser, Esko Ruokonen, Ville Pettilä, Hanno Ulmer, Christian Torgersen, Christian A Schmittinger, Stephan Jakob, Jukka Takala

**Affiliations:** 1 Department of Intensive Care Medicine, Inselspital, Freiburgstrasse, 3010 Bern, Switzerland; 2 Department of Intensive Care, Kuopio University Hospital and Kuopio University, 70211 Kuopio, Finland; 3 Australian and New Zealand Intensive Care Research Centre, Department of EPM, Monash University, 89 Commercial Road, Melbourne 3004, Victoria, Australia; 4 Department of Medical Statistics, Informatics and Health Economics, Innsbruck Medical University, Schöpfstrasse, 6020 Innsbruck, Austria; 5 Department of Anaesthesiology and Critical Care Medicine, Innsbruck Medical University, Anichstrasse 35, 6020 Innsbruck, Austria

## Abstract

**Introduction:**

It is unclear to which level mean arterial blood pressure (MAP) should be increased during septic shock in order to improve outcome. In this study we investigated the association between MAP values of 70 mmHg or higher, vasopressor load, 28-day mortality and disease-related events in septic shock.

**Methods:**

This is a post hoc analysis of data of the control group of a multicenter trial and includes 290 septic shock patients in whom a mean MAP ≥ 70 mmHg could be maintained during shock. Demographic and clinical data, MAP, vasopressor requirements during the shock period, disease-related events and 28-day mortality were documented. Logistic regression models adjusted for the geographic region of the study center, age, presence of chronic arterial hypertension, simplified acute physiology score (SAPS) II and the mean vasopressor load during the shock period was calculated to investigate the association between MAP or MAP quartiles ≥ 70 mmHg and mortality or the frequency and occurrence of disease-related events.

**Results:**

There was no association between MAP or MAP quartiles and mortality or the occurrence of disease-related events. These associations were not influenced by age or pre-existent arterial hypertension (all *P* > 0.05). The mean vasopressor load was associated with mortality (relative risk (RR), 1.83; confidence interval (CI) 95%, 1.4-2.38; *P* < 0.001), the number of disease-related events (*P* < 0.001) and the occurrence of acute circulatory failure (RR, 1.64; CI 95%, 1.28-2.11; *P* < 0.001), metabolic acidosis (RR, 1.79; CI 95%, 1.38-2.32; *P* < 0.001), renal failure (RR, 1.49; CI 95%, 1.17-1.89; *P* = 0.001) and thrombocytopenia (RR, 1.33; CI 95%, 1.06-1.68; *P* = 0.01).

**Conclusions:**

MAP levels of 70 mmHg or higher do not appear to be associated with improved survival in septic shock. Elevating MAP >70 mmHg by augmenting vasopressor dosages may increase mortality. Future trials are needed to identify the lowest acceptable MAP level to ensure tissue perfusion and avoid unnecessary high catecholamine infusions.

## Introduction

Mean arterial blood pressure (MAP) is the driving force for microvascular blood flow and thus an important determinant of tissue perfusion [1]. In its current guidelines [2], the Surviving Sepsis Campaign recommends to maintain a minimum MAP of 65 mmHg in patients with severe sepsis and septic shock. Apart from physiologic knowledge [1], there is weak evidence to support this recommendation. Although the two singlecenter prospective studies specifically investigating MAP levels in septic shock were small, uncontrolled and arbitrarily chose 65 mmHg as their lowest MAP [3,4], a retrospective cohort study supported a MAP of 65 mmHg as the critical level for 30-day survival but was not adjusted for disease severity [5]. The study by Rivers and colleagues targeted a MAP of 65 mmHg in severe sepsis patients but the actual MAP levels were much higher [6]. Therefore, no clinical conclusions about the optimum MAP level can be drawn from this study either. Moreover, in clinical practice, individually different safety limits are often added to the prescribed targets thus resulting in relevantly higher MAP levels than originally prescribed [6-8]. Furthermore, despite the latest recommendations on MAP targets of at least 65 mmHg [2], even the most recent large clinical trials of septic shock have used higher targets and resulted in still substantially higher actual blood pressure levels during sustained administration of catecholamines [8,9].

As vasopressor and inotropic agents are, by definition, required to attain a certain MAP level in septic shock [10], the MAP goal targeted crucially determines the extent of vasopressor or inotropic support. Almost all recommendations of the Surviving Sepsis Campaign regarding the use of vasopressors and inotropes in septic shock are based on catecholamine agents [2]. Whereas it is unquestionable that catecholamines are highly effective drugs to counteract cardiovascular instability [11], they can be associated with disease-related events, particularly at higher dosages [12]. Numerous side effects of catecholamines have been reported for almost all organs and appear particularly devastating on the heart [12]. Hence, finding the lower safe MAP levels could help to reduce excess exposure to exogenous catecholamines and possibly improve outcome.

This *post hoc *analysis of a multicenter trial investigates the influences of MAP levels of 70 mmHg or higher and the vasopressor load on 28-day mortality and disease-related events in septic shock. Our hypothesis was that there would be no association between 28-day mortality and MAP levels of 70 mmHg or higher. Furthermore, we hypothesized that increasing vasopressor dosages may be associated with an increased risk of disease-related events and mortality in septic shock patients.

## Materials and methods

The present study is a *post hoc *analysis of data of an international, multicenter, randomized, double-blind, placebo-controlled clinical trial that investigated the effects of the nitric oxide inhibitor 546C88 on mortality in 797 septic shock patients [13]. The dataset of the control group was provided to the authors by GlaxoSmithKline, UK, the owner of the complete original database.

The original trial was conducted from June 1997 to April 1998. The study protocol was approved by the local ethics committee or institutional review board of each participating center. Written informed consent was obtained from all study patients or their next of kin.

### Inclusion criteria

Patients were included in the original trial based on the following entry criteria: 1) age of 18 years or older; 2) severe sepsis diagnosed less than 72 hours before randomization; 3) septic shock for less than 24 hours defined according to the definitions of the American College of Chest Physicians and the Society of Critical Care Medicine [9] associated with either a MAP of less than 70 mmHg for 30 minutes or more (despite fluid resuscitation) or vasopressor requirement for 30 minutes or more to maintain a MAP of 70 mmHg or higher; 4) pulmonary arterial occlusion pressure of 18 mmHg or less and 8 mmHg or higher if cardiac index less than 5 L/min/m^2^, and adequate fluid resuscitation in the opinion of the investigator; 5) continuous pressure monitoring using systemic and pulmonary arterial catheters; 6) commitment for full life-support measures for the duration of the study; and 7) negative pregnancy test in female patients unless postpartum, previous tubal ligation, hysterectomy, or postmenopausal. After trial inclusion, patients were randomized to a treatment group receiving 546C88 and a control group in which patients received placebo.

Only data of patients allocated to the control group (n = 358) with a mean MAP of 70 mmHg or higher (MAP targeted by the hemodynamic study protocol) during the shock period (n = 290) were included in this *post hoc *analysis. Sixty-eight patients were excluded because their average MAP during the shock period was below the targeted level of 70 mmHg. Characteristics of these patients are shown in Table S1 and S2 of Additional data file 1.

### Clinical and hemodynamic management

Throughout the study, patients were resuscitated according to a strict hemodynamic protocol and local standards of care [13]. Briefly, the hemodynamic protocol included a MAP target of 70 mmHg or higher to be reached by infusion of vasopressors (norepinephrine, dopamine, epinephrine, phenylephrine). Fluid resuscitation was guided at the discretion of the attending physician and was required to attain a pulmonary capillary occlusion pressure of 8 to 18 mmHg if cardiac index was less than 5 L/min/m^2^. Inotropic therapy was instituted to maintain cardiac index of more than 3 L/min/m^2 ^[13].

### Data for the *post hoc *analysis

For the present *post hoc *analysis the following data were retrieved from the original trial's database: demographic data, chronic diseases, details on the infection leading to septic shock, need for surgery or mechanical ventilation, the Simplified Acute Physiology Score (SAPS) II [14] assessed during 24 hours after intensive care unit admission and study randomization, as well as 28-day mortality. MAP values (documented at eight-hourly intervals) were averaged during the shock period (definition see below) after randomization. Based on these average MAP values, study patients were grouped into quartiles. Furthermore, the type and duration of infusion, as well as the mean dosage of catecholamine drugs during the shock period were documented. Because different catecholamine agents were used, the mean vasopressor load was calculated according to a formula suggested by Russell and colleagues [9]: vasopressor load (μg/kg/min) = norepinephrine (μg/kg/min) + dopamine (μg/kg/min/kg/2) + epinephrine (μg/kg/min) + phenylephrine (μg/kg/min/10).

Finally, the original study recorded in a binary fashion the development of the following disease-related events in all patients during their intensive care unit stay, based on the clinical assessment of the investigators: 'cardiac dysrhythmias' (including cardiac arrest), 'acute circulatory failure', 'disseminated intravascular coagulopathy', 'acute hepatic failure', 'metabolic acidosis', 'acute deterioration in mental state' (not due to sedation), 'acute renal failure', 'acute (hypoxemic) respiratory failure', and 'thrombocytopenia'. In addition, the total number of disease-related events (defined as the sum of single disease-related events) was calculated for each study patient.

### Definitions

The duration of shock was defined as the time from study randomization until the patient met all of the following criteria: 1) epinephrine, norepinephrine, phenylephrine, and dobutamine infusion of 0 μg/kg/min; 2) dopamine infusion of 3 μg/kg/min or less; 3) dopexamine infusion of 1 μg/kg/min or less; 4) MAP of 70 mmHg or more [13]. Pre-existence of chronic arterial hypertension was based on contemporary definitions of the World Health Organization. As defined in the original trial [13], disease-related events were considered as events known to be associated with severe sepsis and/or septic shock and considered by the investigator as not having a reasonable possibility of being caused by 546C88 or placebo therapy.

### Study endpoints

The primary endpoint of this *post hoc *analysis was to investigate the association between MAP or MAP quartiles of 70 mmHg or higher and 28-day mortality. Furthermore, we sought to evaluate whether this association was influenced by age, pre-existent arterial hypertension or the mean vasopressor load. The secondary endpoint was to investigate the association between MAP or MAP quartiles of 70 mmHg or higher and the occurrence of disease-related events. Again the influence of age, pre-existent arterial hypertension and the mean vasopressor load on these associations was evaluated.

### Statistical analysis

The SPSS software program was used for statistical analysis (SPSS 15.0; SPSS Inc, Chicago, IL, USA). Kolmogorov-Smirnov tests were applied to check for normality distribution of data which was approximately fulfilled by all variables except the mean vasopressor load. This variable underwent *ln*-transformation and subsequently showed normal distribution.

Descriptive statistical methods were used to present study variables. For comparisons between survivors and non-survivors, Student's *t*-tests and Fisher's Exact tests were applied, as appropriate. Binary logistic regression models were used to answer the primary and secondary study endpoints. These models included either 28-day mortality or the occurrence of disease-related events as the dependent variable. MAP (linear) or MAP quartiles (categorical, applying simple-first comparisons) were entered as covariates. In order to adjust for disease severity and therapeutic differences between geographic regions as well as to evaluate the influence of age, pre-existent arterial hypertension and the mean vasopressor load, all logistic regression models included the SAPS II (excluding the systolic arterial blood pressure count) assessed during the first 24 hours after randomization, the geographic region of the study center, age, presence of chronic arterial hypertension and the mean vasopressor load during the shock period as covariates. None of these covariates showed relevant colinearity between each other or MAP (all, Spearman rank correlation coefficient less than 0.35). To evaluate the association between the total number of disease-related events, MAP and the mean vasopressor load, linear regression models with the same covariates as the above mentioned logistic regression model were calculated.

In an earlier sepsis population [15] and other patient groups [16,17], heart rate was indirectly or directly correlated with mortality, so the association between the mean heart rate during the shock period and 28-day mortality was evaluated in this study population using the same adjusted logistic regression model. As the hemodynamic protocol of the original trial did not include heart rate targets, all patients allocated to the control group (n = 358) were included into the latter model.

For all comparisons and models, a *P*-value less than 0.05 was assumed to indicate statistical significance. Throughout the manuscript data are presented as mean values ± standard deviation, if not otherwise indicated.

## Results

Tables S1 and S2 of the Additional data file 2 present demographic and clinical data of the study population. The leading causes of death (by day 90) were multiple organ dysfunction syndrome (n = 61; 45.9%), refractory shock (n = 23; 17.3%), respiratory failure (n = 20; 15%), and miscellaneous (n = 29; 21.8%). Non-survivors were older, acquired infection more often in the intensive care unit, had a higher SAPS II after randomization, more disease-related events (except for mental deterioration) and required more and higher vasopressor dosages than survivors.

**Figure 1:**
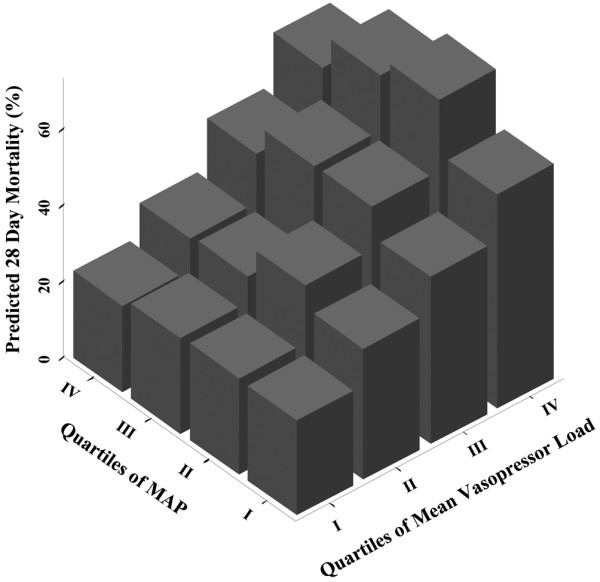
28-day mortality by MAP and mean vasopressor load quartiles as predicted by the adjusted logistic regression model. Mean arterial blood pressure (MAP) quartile I = 70 to 74.3 mmHg; MAP quartile II = 74.3 to 77.8 mmHg; MAP quartile III = 77.8 to 82.1 mmHg; MAP quartile IV = 82.1 to 99.7 mmHg.

There was no association between MAP quartiles and 28-day mortality. As the only covariates SAPS II and the mean vasopressor load showed a significant association with mortality in the adjusted logistic regression model [Table S3 in Additional data file 2]. The predicted 28-day mortality by MAP and mean vasopressor load quartiles is displayed in Figure 1. When introducing MAP (mmHg) as a linear variable instead of MAP quartiles into the model it was not associated with death at day 28 (Wald, 0.054; relative risk (RR), 0.99; 95% confidence interval (CI), 0.95 to 1.04; *P *= 0.82).

**Figure 2:**
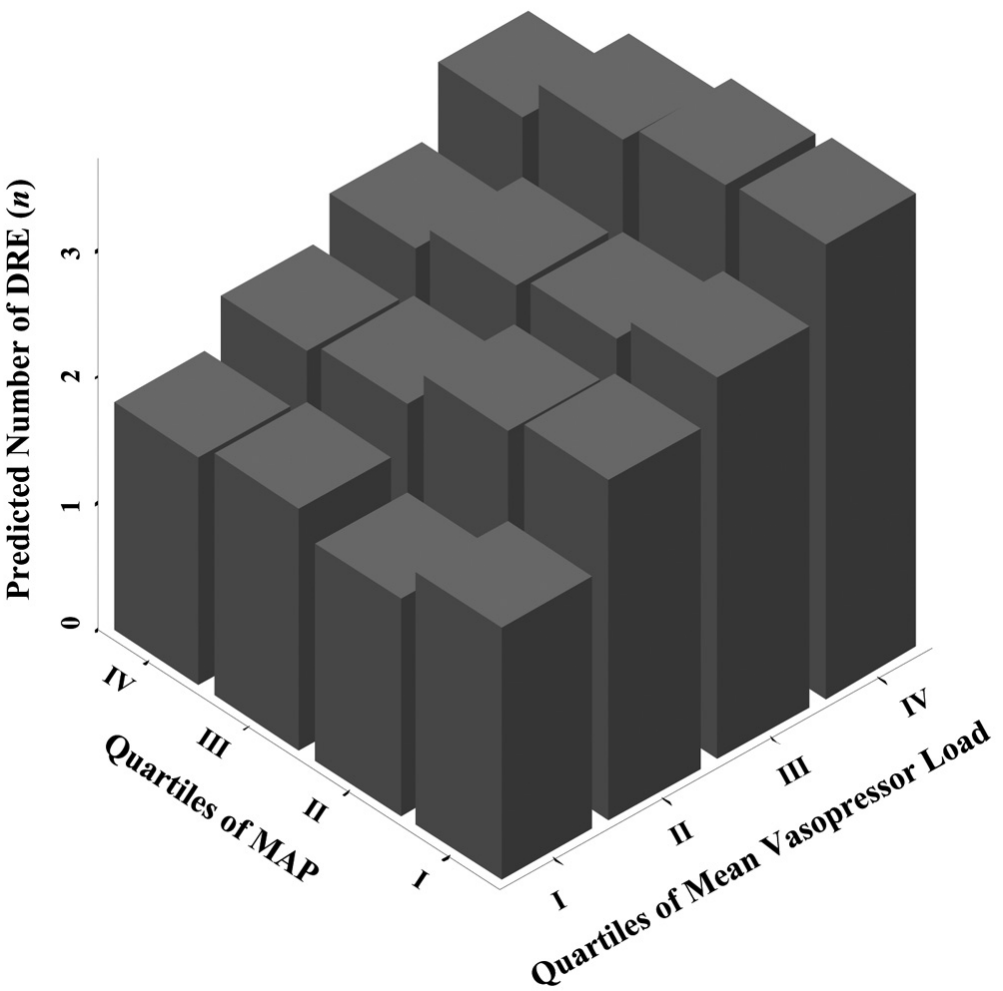
Number of DRE by MAP and mean vasopressor load quartiles as predicted by the adjusted logistic regression model. Mean arterial blood pressure (MAP) quartile I = 70 to 74.3 mmHg; MAP quartile II = 74.3 to 77.8 mmHg; MAP quartile III = 77.8 to 82.1 mmHg; MAP quartile IV = 82.1 to 99.7 mmHg. DRE = disease-related events.

MAP or MAP quartiles were not associated with the total number of disease-related events (linear regression model; MAP: standardized Beta-Coefficient, -0.052; *P *= 0.36; MAP quartiles: standardized Beta-Coefficient, -0.035; *P *= 0.55) or the occurrence of any single disease-related event. These associations were not influenced by age or pre-existent arterial hypertension. However, the mean vasopressor load was significantly associated with the total number of disease-related events (standardized Beta-Coefficient, 0.225; *P *< 0.001). Figure 2 presents the predicted number of total disease-related events by MAP and mean vasopressor load quartiles as predicted by the adjusted logistic regression model. The mean vasopressor load (*per ln unit*) was associated with the occurrence of acute circulatory failure (RR, 1.64; 95% CI, 1.28 to 2.11; *P *< 0.001), metabolic acidosis (RR, 1.79; 95% CI, 1.38 to 2.32; *P *< 0.001), renal failure (RR, 1.49; 95% CI, 1.17 to 1.89; *P *= 0.001) and thrombocytopenia (RR, 1.33; 95% CI, 1.06 to 1.68; *P *= 0.01) in single adjusted logistic regression models. Study patients still had a significantly lower mean and maximum vasopressor load during the shock period when compared with the 68 patients excluded from the analysis (mean vasopressor load, 0.64 ± 1.92 *vs. *2.31 ± 6.56 μg/kg/min, *P *= 0.003; maximum vasopressor load, 1.19 ± 3.54 *vs. *3.06 ± 7.4 μg/kg/min, *P *= 0.01) [Figure S1 in Additional data file 1].

The mean heart rate during the shock period was associated with 28-day mortality in the adjusted logistic regression model (RR 1.029; 95% CI, 1.01 to 1.047; *P *< 0.001) [Table S3 in Additional data file 1]. Mean heart rates in the highest sixtile (>122 bpm) were associated with a significantly higher 28-day mortality than heart rates in the lowest sixtile (<92 bpm). Again, the mean vasopressor load revealed the strongest association with 28-day mortality.

## Discussion

The results of this *post hoc *analysis confirmed our study hypothesis that MAP levels exceeding 70 mmHg were not associated with 28-day mortality or the occurrence of disease-related events in patients with septic shock. In contrast, any increase of MAP over 70 mmHg achieved by an increase of vasopressor dosages appears to be associated with the number of disease-related events and mortality.

A limitation of our study is that analysed data were collected more than a decade ago and it can be argued that hemodynamic management of septic shock has changed since then. Specifically, the recent Surviving Sepsis Campaign recommended maintaining a minimum MAP of 65 mmHg as opposed to 70 mmHg in this trial. However, the most recent large clinical trials in septic shock patients suggest that recommendations to tolerate lower MAP levels have not become standard in clinical practice. Indeed, in the Vasopressin in Septic Shock and Catecholamines in Septic Shock CATS trials, the mean MAP achieved during septic shock was about 75 to 80 mmHg (two standard deviations up to 110 mmHg) [8] and about 75 (two standard deviations up to 90 to 100 mmHg) [9], respectively. Similarly, the mean norepinephrine dose infused during the first two days after randomization was about 1.1 μg/kg/min (two standard deviations up to 5 μg/kg/min) in the CATS trial [8]. Infusion of even higher catecholamine dosages in critically ill patients with septic shock have lately been reported by others [18]. Furthermore, a recent clinical study has suggested that targeting higher MAP by increasing norepinephrine resulted in an increase in global oxygen delivery and tissue oxygenation [19]. Moreover, hemodynamic goals in our study patients other than MAP were comparable with current recommendations [2,13]. Accordingly, the results of our analysis appear to be clinically relevant still today.

It is important to note that all statistical models in this analysis were adjusted for factors commonly presumed to influence the association between MAP and mortality. An important covariate was disease severity as assessed by SAPS II, which should have unmasked gross influences of the underlying disease on the association between MAP and mortality. Nonetheless, it is conceivable that although SAPS II is a reliable measure of disease severity and excellent predictor of mortality [14], it may not reflect the true extent of cardiovascular failure and other cofactors that impact on 28-day mortality. Furthermore, despite including 290 patients into the analysis, the sample sizes in MAP quartiles may have been too small to uncover statistical significance. Nonetheless, given a RR ratio of 0.99 (95% CI, 0.95 to 1.04) per mmHg MAP increase for death at day 28, it is unlikely that significance would have been reached had more patients been included.

This analysis included 290 of the 358 patients who were included in the control group of the original trial. Sixty-eight patients had to be excluded because the goal to maintain a MAP of at least 70 mmHg during the shock period could not be achieved. As the hemodynamic protocol of the original trial strictly required a MAP of 70 mmHg or higher, it must be assumed that patients who did not attain this MAP level were either too sick to achieve the target (vasopressor-resistant hypotension) or had undergone violations of the hemodynamic protocol. Both options preclude meaningful comparisons between the 68 patients excluded and the current study population as well as the evaluation of the association between MAP levels less than 70 mmHg and mortality in septic shock. Accordingly, although our results indicate that MAP levels of 70 mmHg or higher are not associated with improved outcome in septic shock patients, they cannot prove whether a MAP of 70 mmHg is optimal for survival or if the critical MAP level is lower than that. We therefore hypothesize that identification of a critical MAP level lower than 70 mmHg could further decrease vasopressor exposure, the frequency of disease-related events and mortality in septic shock patients. This hypothesis should be tested in future prospective studies.

The present data, which were collected from patients treated in 124 intensive care units worldwide, are in accordance with results of previous single-center studies. Two prospective studies evaluating the effects of different MAP levels on tissue perfusion and renal function in septic shock observed that increasing MAP from 65 to 85 mmHg did not improve systemic oxygen metabolism, skin microcirculatory blood flow, splanchnic perfusion nor renal function [3,4]. Similar to our results, relevant increases of norepinephrine were required to increase MAP from 65 to 85 mmHg in both studies. Two retrospective studies applying similar statistical models observed that the critical MAP for 30 or 28-day mortality in septic shock and sepsis was 65 [5] and 60 mmHg [20], respectively.

Neither age nor pre-existent arterial hypertension relevantly influenced the association between MAP and 28-day mortality or the occurrence of disease-related events including renal failure. However, considering the wide CIs of the influence of pre-existent hypertension on the association between MAP and mortality, it is possible that the present analysis yielded false-negative results. Based on current physiologic and pathophysiologic understanding [1], it would be expected that in elderly and/or chronic hypertensive patients organ autoregulation curves, particularly renal, are shifted to the right and higher MAP levels needed to preserve organ function and ensure survival. Preliminary results in another sepsis population similarly suggest that neither age nor chronic arterial hypertension has a clinically relevant impact on the association between MAP and mortality [20].

Metabolic acidosis related to catecholamine therapy has been typically observed during epinephrine infusion and may originate from epinephrine-related acceleration of metabolism and/or induction of tissue hypoperfusion [21,22]. In earlier studies, catecholamines have repeatedly been associated with disease-related events on cardiac function ranging from ischemia to myocardial stunning and apoptosis [12]. Tachycardia is a particularly common and well-known side effect of catecholamine therapy [12]. The significant association between heart rate during the shock period and 28-day mortality in this patient population confirms the results of an earlier prospective observational study in 48 septic shock patients [15]. Whereas beneficial effects of vasopressors have been reported [11] and adrenergic vasopressors are recommended as first-line agents in septic shock [2], we observed an independent association between the mean vasopressor load during shock and both the development of disease-related events as well as death at day 28. Two recent studies reporting adverse effects of catecholamine vasopressors on organ function [23] and mortality [24] in septic shock support our results. Furthermore, the findings of the present analysis are in line with earlier data suggesting harmful effects of excess catecholamine exposition in general critically ill patient populations. For example, Boldt and colleagues showed that circulating plasma levels of catecholamines were higher in non-surviving when compared with surviving surgical intensive care unit patients [25]. A randomized trial that investigated the outcome effects of supranormal oxygen delivery in a diverse group of critically ill patients reported higher in-hospital mortality in patients receiving liberal catecholamine therapy than in control patients exposed to standard care [26].

Important limitations must be considered when interpreting the results of this study. First, and probably most importantly, mean values of punctually instead of continuously recorded MAPs were analysed. Thus, the true course of MAP may have been under- or over-estimated. Additionally, it is possible that some patients changed between MAP quartiles during the shock period but were eventually grouped into one quartile based on their average MAP. Second, as the original study was performed in the late 1990s the definition of some disease-related events does not correspond to current recommendations. This is particularly relevant for the definition of renal failure [27] and disseminated intravascular coagulation [28], which has recently been newly defined based on international consensus. Furthermore, the occurrence of disease-related events was documented during the intensive care unit stay after study randomization. Although more than half of non-surviving study patients did not achieve shock resolution and developed disease-related events during the evaluated shock period, it is possible that some disease-related events occurred either after shock resolution or during a renewed shock episode during which MAP and the mean vasopressor load were not evaluated. When drawing clinical conclusions from our results caution is warranted because the MAP quartiles analysed were retrospectively defined and can not be considered as treatment goals. Finally, it must be considered that this *post hoc *analysis was performed in an uncontrolled patient cohort, and its results must not be considered to have the same validity as those of a randomized, controlled trial.

## Conclusions

MAP levels of 70 mmHg or higher do not appear to be associated with improved survival in septic shock. However, augmenting vasopressor dosages to elevate MAP to more than 70 mmHg may increase mortality. Future trials are needed to identify the lowest acceptable MAP level to ensure tissue perfusion and avoid unnecessary high catecholamine infusions.

## Key messages

• MAP levels of 70 mmHg or higher do not appear to be associated with improved survival in septic shock.

• Augmenting vasopressor dosages to elevate MAP to more than 70 mmHg may increase mortality.

• Future trials are needed to identify the lowest acceptable MAP level to ensure tissue perfusion and avoid unnecessary high catecholamine infusions.

## Abbreviations

CI: confidence interval; MAP: mean arterial blood pressure; RR: relative risk; SAPS: Simplified Acute Physiology Score.

## Competing interests

The authors declare that they have no competing interests.

## Authors' contributions

MWD made substantial contributions to conception and design of the study, acquired, analysed and interpreted data, drafted the manuscript and gave final approval of the version to be published. ER made substantial contributions to conception and design of the study, interpreted data, revised the manuscript for important intellectual content and gave final approval of the version to be published. VP made substantial contributions to conception and design of the study, interpreted data, revised the manuscript for important intellectual content and gave final approval of the version to be published. HU analysed and interpreted the data, revised the manuscript for important intellectual content and gave final approval of the version to be published. CT acquired and interpreted data, revised the manuscript for important intellectual content and gave final approval of the version to be published. CAS acquired and interpreted data, revised the manuscript for important intellectual content and gave final approval of the version to be published. SJ made substantial contributions to conception and design of the study, interpreted data, revised the manuscript for important intellectual content and gave final approval of the version to be published. JT made substantial contributions to conception and design of the study, interpreted data, revised the manuscript for important intellectual content and gave final approval of the version to be published.

## Supplementary Material

Additional file 1 A Word file containing three tables and one figure. Table S1 is a table that lists the characteristics of the 68 excluded patients. Table S2 is a table that lists the disease-related events and vasopressor support during the shock period in the 68 excluded patients. Table S3 is a table that lists the association between heart rate during septic shock and 28-day mortality. The figure presents the vasopressor load in study patients with a mean arterial blood pressure (MAP) of less than 70 mmHg during the shock period (n = 68; mean vasopressor load 2.31 ± 6.56 μg/kg/min) compared with the mean vasopressor load in study patients with a MAP of more than 70 mmHg during the shock period (n = 290; mean vasopressor load 0.64 ± 1.92 μg/kg/min, reference line). Click here for file

Additional file 2 A Word file containing three tables. Table S1 is a table that lists the characteristics of the study population. Table S2 is a table that lists disease-related events and vasopressor support during the shock period. Table S3 is a table that lists the adjusted logistic regression model to evaluate the association between mean arterial blood pressure (MAP), mean vasopressor load and 28-day mortality. Click here for file
