# Supplementary material for: Association of arterial blood pressure and vasopressor load with septic shock mortality: a post hoc analysis of a multicenter trial
Source: Crit Care. 2009 Nov 16;13(6):R181. doi: 10.1186/cc8167 (PMC2811945; doi:10.1186/cc8167)
Supplement: Additional file 1 — A Word file containing three tables and one figure. Table S1 is a table that lists the characteristics of the 68 excluded patients. Table S2 is a table that lists the disease-related events and vasopressor support during the shock period in the 68 excluded patients. Table S3 is a table that lists the association between heart rate during septic shock and 28-day mortality. The figure presents the vasopressor load in study patients with a mean arterial blood pressure (MAP) of less than 70 mmHg during the shock period (n = 68; mean vasopressor load 2.31 ± 6.56 μg/kg/min) compared with the mean vasopressor load in study patients with a MAP of more than 70 mmHg during the shock period (n = 290; mean vasopressor load 0.64 ± 1.92 μg/kg/min, reference line). [file cc8167-S1.DOC]

**Methods and Statistical Analysis**

For the presented logistic regression analysis, the HR during the shock period after study randomization was averaged and grouped into sixtiles. The statistical model included 28 day mortality as the dependent variable and HR sixtiles (categorical, applying simple-first comparisons) as covariates. In order to adjust for disease severity and therapeutic differences between geographic regions as well as to evaluate the influence of age, pre-existent arterial hypertension and the mean vasopressor load, the logistic regression model included the SAPS II (excl. the heart rate count) assessed during the first 24 hours after randomization, the geographic region of the study center, age, presence of chronic arterial hypertension and the mean vasopressor load during the shock period as covariates. Since the hemodynamic protocol of the original trial did not include heart rate targets, all patients allocated to the control group (*n*=358) were included into the model.

**Reference Line**

**Figure 1.**

Vasopressor Load in Study Patients with a MAP <70 mmHg during the Shock Period (*n*=68; mean vasopressor load 2.31±6.56 µg/kg/min) compared with the Mean Vasopressor Load in Study Patients with a MAP >70 mmHg during the Shock Period (*n*=290; mean vasopressor load 0.64±1.92 µg/kg/min, reference line).
