# Supplementary material for: Association of arterial blood pressure and vasopressor load with septic shock mortality: a post hoc analysis of a multicenter trial
Source: Crit Care. 2009 Nov 16;13(6):R181. doi: 10.1186/cc8167 (PMC2811945; doi:10.1186/cc8167)
Supplement: Supplementary file 2 [file cc8167-S2.DOC]

Table 1. Characteristics of the Study Population

		Total	Survivors	Non-Survivors	*p* -value
n		290	168	122	
**Age**	*(years)*	60 ± 15	58 ± 15	63 ± 14	0.02*
**Male Sex**	*n (%)*	178 (61.4)	98 (58.3)	80 (65.6)	0.22
**BMI**	*(kg/m²)*	27 ± 6	27 ± 6	27 ± 6	0.8
**Chronic Diseases**	*n (%)*				
Arterial Hypertension		91 (31.4)	48 (28.6)	43 (35.2)	0.25
Alcoholism		35 (12.1)	16 (9.5)	19 (15.6)	0.14
Diabetes mellitus		39 (13.4)	19 (11.3)	20 (16.4)	0.23
Chronic Pulmonary Disease		41 (14.1)	22 (13.1)	19 (15.6)	0.61
Cancer		33 (11.4)	20 (11.9)	13 (10.7)	0.85
Other Chronic Diseases		157 (54.1)	84 (50)	73 (59.8)	0.12
**Primary Site of Infection**	*n (%)*				
Respiratory Tract		100 (34.5)	54 (32.1)	46 (37.7)	0.38
Intra-abdominal		59 (20.3)	35 (20.8)	24 (19.7)	0.88
Gastrointestinal Tract		27 (9.3)	15 (8.9)	12 (9.8)	0.84
Urinary Tract		16 (5.5)	10 (6)	6 (4.9)	0.8
Others/missing		88 (30.3)	54 (32.1)	34 (27.9)	0.52
**Origin of Infection**	*n (%)*				
Community		161 (55.5)	99 (58.9)	62 (50.8)	0.19
Hospital		90 (31)	51 (30.4)	39 (32)	0.8
Intensive Care Unit		21 (7.2)	7 (4.2)	14 (11.5)	0.02*
Unknown		18 (6.2)	11 (6.5)	7 (5.7)	0.81
**Infection Confirmed**	*n (%)*	212 (73.1)	123 (73.2)	89 (73)	1
**Need for Surgery**	*n (%)*	147 (50.7)	85 (50.5)	62 (59.9)	1
**Need for MV**	*n (%)*	279 (96.2)	158 (94)	121 (99.2)	0.05
**SAPS II**	*(points)*				
after ICU admission		48 ± 14	47 ± 13	50 ± 15	0.05
after randomization		50 ± 14	47 ± 12	53 ± 15	<0.001*
**Duration of Shock**	*(hours)*	107 ± 98	98 ± 87	118 ± 111	0.22
**28 Day Mortality**	*n (%)*	122 (42.1)	...	...	
**90 Day Mortality**	*n (%)*	133 (45.9)	...	...	

BMI, body mass index; MV, mechanical ventilation; SAPS, simplified acute physiology score; ICU, intensive care unit; ..., not applicable; *, significant difference between survivors and non-survivors.
Data are given as mean values±SD if not otherwise indicated.

Table 2. Disease-Related Events and Vasopressor Support During the Shock Period

		Total	Survivors	Non-Survivors	*p* -value
n		290	168	122	
**Disease-Related Events**	*n (%)*				
Cardiac Dysrhythmias		111 (38.3)	43 (25.6)	68 (55.7)	<0.001*
Acute Circulatory Failure		103 (35.5)	27 (16.1)	76 (62.3)	<0.001*
DIC		42 (14.5)	13 (7.7)	29 (23.8)	<0.001*
Acute Hepatic Failure		46 (15.9)	13 (7.7)	33 (27)	<0.001*
Metabolic Acidosis		84 (29)	22 (13.1)	62 (50.8)	<0.001*
Acute Mental Deterioration		52 (17.9)	25 (14.9)	27 (22.1)	0.12
Acute Renal Failure		105 (36.2)	29 (17.3)	76 (62.3)	<0.001*
Acute Respiratory Failure		110 (37.9)	43 (25.6)	67 (54.9)	<0.001*
Thrombocytopenia		102 (35.2)	49 (29.2)	53 (43.4)	0.01*
**Vasopressor Support**					
Number of Catecholamines	*n*	2.2 ± 0.8	2.0 ± 0.8	2.2 ± 0.8	0.004*
Mean Vasopressor Load	*(µg/kg/min)*	0.64 ± 1.92	0.35 ± 0.98	1.03 ± 2.67	<0.001*
Maximum Vasopressor Load	*(µg/kg/min)*	1.19 ± 3.53	0.66 ± 2.18	1.19 ± 4.72	<0.001*

DIC, disseminated intravascular coagulopathy; *, significant difference between survivors and non-survivors.
Data are given as mean values±SD if not otherwise indicated.

Table 3. Adjusted Logistic Regression Model to Evaluate the Association between MAP, Mean Vasopressor Load and 28 Day Mortality

	Variable Type	Unit	Wald	RR	95% CI	*p* -value
**Mean Vasopressor Load**	Continuous	*(per ln unit)*	19.993	1.83	1.4-2.38	<0.001*
**SAPS II (excl. SAP count)**	Continuous	*(points)*	7.249	1.03	1.01-1.05	0.007*
**Age**	Continuous	*(years)*	3.002	1.02	0.99-1.04	0.08
**Geographic Region**	Categorical		0.998	1.02	0.99-1.05	0.32
**MAP Quartiles**	Categorical					
MAP Quartile I (70-74.3 mmHg; n=73)			0.76	*Reference*		0.86
MAP Quartile II (74.3-77.8 mmHg; n=72)			0.645	1.34	0.65-2.76	0.42
MAP Quartile III (77.8-82.1 mmHg; n=73)			0.234	1.19	0.58-2.45	0.63
MAP Quartile IV (>82.1 mmHg; n=72)			0.023	1.06	0.5-2.25	0.88
**Arterial Hypertension**	Categorical		0.001	0.99	0.56-1.76	0.98

MAP, mean arterial blood pressure; RR, relative risk; CI, confidence interval; SAPS, Simplified Acute Physiology Score; SAP, systolic arterial blood pressure; *, significant association with 28 day Mortality.
Variables are ranked (top to bottom) according to the value of the Wald statistics.
